# Clinical evaluation of the Magicplex Sepsis Real-time Test (Seegene) to detect Candida DNA in pediatric patients

**DOI:** 10.1186/cc11729

**Published:** 2012-11-14

**Authors:** J Serra, E Rosello, C Figueras, M Pujol, Y Peña, P Céspedes, JL Dapena, C Díaz-Heredia, MG Codina, A Andreu

**Affiliations:** 1 Hospital Vall d'hebron, Barcelona, Spain

## Background

Invasive candidiasis is an important cause of morbidity and mortality among hospitalized patients. A rapid diagnosis is extremely important for a successful outcome. Standard diagnostic methods (culture and direct examination) often lack a satisfying sensitivity and require a long growth period. The aim of the study was to assess the performance of a new real-time PCR multiplex technique to rapidly detect Candida DNA in clinical samples compared with culture-based methods.

## Methods

Between November 2011 and May 2012, 142 blood samples from 71 pediatric critical or hematological patients were studied both by culture and PCR. The Magicplex Sepsis Real-time Test (Magicplex™ system; Seegene) was used to detect *Candida albicans*, *Candida parapsilosis*, *Candida glabrata*, *Candida tropicalis *and *Candida krusei*. Prior to DNA extraction (EZ1 automatic system; Qiagen) at least 1 ml of blood sample was inoculated into a VersaTREK REDOX1 bottle, which was incubated at 37°C for ≥3 hours. The PCR technique was performed according to the manufacturer's instructions. Culture-based methods were conduced simultaneously in all specimens using the BacT/ALERT system (BioMérieux).

## Results

A total of 131 of the 142 samples were both culture and PCR negative. Nine blood samples (belonging to eight patients) were positive by PCR, two of them were also positive by culture and the remaining seven were not; two of the unfitting samples (28.5%) belonged to patients with other multiple positive cultures for *Candida *spp. (urine and/or catheter devices); the others belonged to patients with high clinical suspicion and risk factors for invasive candidiasis. On the other hand, two culture-proven and PCR-negative samples were considered false negative results. Blood culture allowed the diagnosis of four patients (two of them were also positive for PCR), while the evaluated PCR technique permitted the detection of six additional cases (one of them with two positive samples).

## Conclusion

In this study the evaluated PCR system provides a rapid technique for Candida detection. It can be considered an effective diagnostic tool for diagnosing invasive candidiasis along with conventional cultures.

